# Quantitation, networking, and function of protein phosphorylation in plant cell

**DOI:** 10.3389/fpls.2012.00302

**Published:** 2013-01-08

**Authors:** Lin Zhu, Ning Li

**Affiliations:** Division of Life Science, The Hong Kong University of Science and TechnologyHong Kong, China

**Keywords:** SILIA, AQUIP, plant, quantitative proteomics, post-translational modification, cell signaling and regulation, mass spectrometry-based interactomics

## Abstract

Protein phosphorylation is one of the most important post-translational modifications (PTMs) as it participates in regulating various cellular processes and biological functions. It is therefore crucial to identify phosphorylated proteins to construct a phosphor-relay network, and eventually to understand the underlying molecular regulatory mechanism in response to both internal and external stimuli. The changes in phosphorylation status at these novel phosphosites can be accurately measured using a ^15^N-stable isotopic labeling in Arabidopsis (SILIA) quantitative proteomic approach in a high-throughput manner. One of the unique characteristics of the SILIA quantitative phosphoproteomic approach is the preservation of native PTM status on protein during the entire peptide preparation procedure. Evolved from SILIA is another quantitative PTM proteomic approach, AQUIP (absolute quantitation of isoforms of post-translationally modified proteins), which was developed by combining the advantages of targeted proteomics with SILIA. Bioinformatics-based phosphorylation site prediction coupled with an MS-based *in vitro* kinase assay is an additional way to extend the capability of phosphosite identification from the total cellular protein. The combined use of SILIA and AQUIP provides a novel strategy for molecular systems biological study and for investigation of *in vivo* biological functions of these phosphoprotein isoforms and combinatorial codes of PTMs.

## INTRODUCTION

As one of hundreds of known post-translational modifications (PTM), protein phosphorylation participates in almost every regulatory event known to us in cellular process and biological function (Krishna and Wold, 1993; Hunter, 2000). It is therefore regarded as one of the most important regulatory PTM events. Understanding of the regulation of protein phosphorylation in response to environmental cues/stimuli provides novel insights into how phosphor-relay-mediated signals are transduced and regulated within a plant cell. Mass spectrometry-based phosphoproteomics has emerged as a powerful and high-throughput approach in profiling phosphoproteins participating in cellular processes and signaling regulation (Schmelzle and White, 2006; Schulze, 2010). Recently, various quantitative proteomics strategies have been developed to gain insight into the phosphoprotein dynamics, including label-free, chemical or stable-isotope labeling and corresponding statistical assessment (Schulze and Usadel, 2010). *In vivo* metabolic incorporation of stable isotopes, particularly the heavy nitrogen (^15^N), is firstly described in 1999 (Oda et al., 1999) and has emerged as one of the favorite strategies given the autotrophic nature of plants (Dunkley et al., 2004; Gevaert et al., 2008; Gouw et al., 2010; Guo and Li, 2011; Arsova et al., 2012). Examples are the stable isotope labeling by/with amino acids in cell culture (SILAC; Ong et al., 2002) and the stable isotope labeling in planta (SILIP; Schaff et al., 2008). Based on these protocols, treated and untreated plants are differentially labeled with ^14^N- or ^15^N-coded salt, respectively, with reciprocal repeats. Consequently, proteins from each group of plants are differentially labeled and are mixed at ratios of 1:1–1.5 (^14^N:^15^N) depending on the actual incorporation rate before a mixture of peptides are processed (Guo and Li, 2011). This early protein mixing step is supposed to exclude the variation resulting from peptide preparation, separation, and MS analysis processes (Gevaert et al., 2008). The absolute quantification of proteins (AQUA; Gerber et al., 2003) was introduced in 2003 by Steven Gygi’s group. Heavy isotope-labeled peptides are used by AQUA method as the internal standard, added preferentially as early as possible in the analytical process. Multiplexed absolute quantification was achieved by constructing a recombinant gene that concatenates different tryptic peptides to be quantified (Beynon et al., 2005; Pratt et al., 2006).

As there are already a number of excellent reviews covering the developments and perspectives in quantitative proteomics (Aebersold and Mann, 2003; Ong and Mann, 2005; Bantscheff et al., 2007; Schulze and Usadel, 2010) as well as plant proteomics (Gerber et al., 2003; Chen and Harmon, 2006; Thelen and Peck, 2007; Kota and Goshe, 2011; Arsova et al., 2012), this review will focus on the application of ^15^N stable isotope-based quantitative and differential PTM proteomics in identification of important PTM protein components during cellular processes and on the investigation of their *in vivo* functions in plant. The ^15^N-stable isotope labeling in Arabidopsis (SILIA)-based quantitative proteomic protocol is first applied onto Arabidopsis grown on a solid-medium (Guo and Li, 2011). Consequently, the successfully identified phosphosites are further investigated by bioinformatics-prediction and *in vitro* kinase assays in combination with mutant kinase extracts and quantitative methods such as the isobaric tags for relative and absolute quantitation (iTRAQ; Ross et al., 2004) so that these phosphopeptides can be first validated and a subgroup of highly interesting phosphorylation sites are selected for the following *in vivo* quantitation using the absolute quantitation of isoforms of post-translationally modified proteins (AQUIP) approach (Li et al., 2012). AQUIP was developed from the targeted and quantitative proteomics by combining the advantages of AQUA (Gerber et al., 2003) and the protein standard absolute quantification strategy (PSAQ; Brun et al., 2007; Lebert et al., 2011) with SILIA. Finally, at the end of the integrated *in vitro* and *in vivo* quantitative PTM proteomics, the biological function studies are performed on the group of highly selected phosphosites and phosphoproteins to unravel their molecular, cellular, and biological roles in plants.

## SILIA-BASED QUANTITATIVE PHOSPHOPROTEOMICS

Previous metabolic labeling experiments were mostly performed in aqueous solutions in the form of cell suspension cultures (SILAC; Engelsberger et al., 2006; Benschop et al., 2007) or seedlings suspended in liquid media (Dunkley et al., 2004; Huttlin et al., 2007; Nelson et al., 2007). The labeling was further performed on plants either grown in hydroponic solutions (HILEP, the hydroponic isotope labeling of entire plants; Bindschedler et al., 2008; Hebeler et al., 2008) or in soil (SILIP; Schaff et al., 2008). In the case of SILIA, a general metabolic-labeling strategy was designed for agar-based plant growth, which is frequently used in molecular genetic screens and/or physiological studies. SILIA-medium was designed to allow the ^15^N as the only nitrogen source for Arabidopsis growth and to maximize the incorporation rate (Guo and Li, 2011). Another major improvement in SILIA-based quantitative PTM proteomics is the use of urea-based protein denaturing buffer (UEB), which provides a fully denaturing condition to inhibit protease-mediated protein degradation during protein extraction/separation processes and, especially, to prevent non-specific phosphorylation/dephosphorylation events from occurring in protein extracts. A detailed workflow of the SILIA-based quantitative proteomics has been described in an earlier publication (Guo and Li, 2011). As data generated by quantitative proteomics methods (e.g., iTRAQ and SILIA) usually show relative changes (i.e., fold change) in phosphorylation among different conditions, statistical methods are needed to confirm that there is a significant difference between the degrees of phosphorylation of two conditions (de la Fuente van Bentem et al., 2008). Both forward and reciprocal experiments are usually repeated two to four times to obtain a set of satisfactory quantitative phosphoprotein-profiling data.

## *IN VITRO* KINASE ASSAY TO VALIDATE THE MS-IDENTIFIED AND BIOINFORMATICS-PREDICTED PHOSPHOSITES

Non-biased phosphoprotein profiling performed by SILIA provides valuable quantitative information about protein phosphorylation changes in response to external or internal stimuli in plants. As MS-identification of phosphopeptides is routinely evaluated with a false discovery rate (FDR), *in vitro* phosphorylation validation is usually required to be performed on a group of randomly selected phosphopeptides before they are chosen as candidates for further *in vivo* quantitation and a long-term functional study. Secondly, although substantial advancements have been made in MS technology and bioinformatics software during the discovery of phosphosites, the number of MS-identified phosphopeptides is still far below the theoretically predicted number (de la Fuente van Bentem et al., 2008; Heazlewood et al., 2008; Durek et al., 2010). Thus, prediction of putative phosphosites from inducible phosphorylation sites using bioinformatics is expected to increase the probability of identifying additional phosphosites.

The amino acid sequence around a phosphosite serves as a structural feature for kinase’s substrate recognition (Jorgensen and Linding, 2008; Mok et al., 2010). Because the amino acid sequence surrounding the phosphosite may be highly conserved among various substrates, a short stretch (9–21 amino acid-long) oligopeptide deduced from the primary sequence of an identified phosphosite, is employed to BLAST against the non-redundant plant protein sequence database (Arabidopsis in this case; Li et al., 2009). The alignment leads to the identification of phosphorylation motif. Synthetic peptides are then made according to the predicted phosphosites and fused to a hexa-His tag at N-terminus to facilitate substrate peptide purification following kinase assay. Huber and colleagues have demonstrated a case, in which a highly conserved phosphorylation site was established among ACC synthase (a key enzyme in ethylene biosynthesis) isomers using *in vitro* kinase assay and the calmodulin-dependent protein kinase (CDPK) was found to be responsible for the phosphorylation of this site (Hernandez et al., 2004).

**FIGURE 1:**
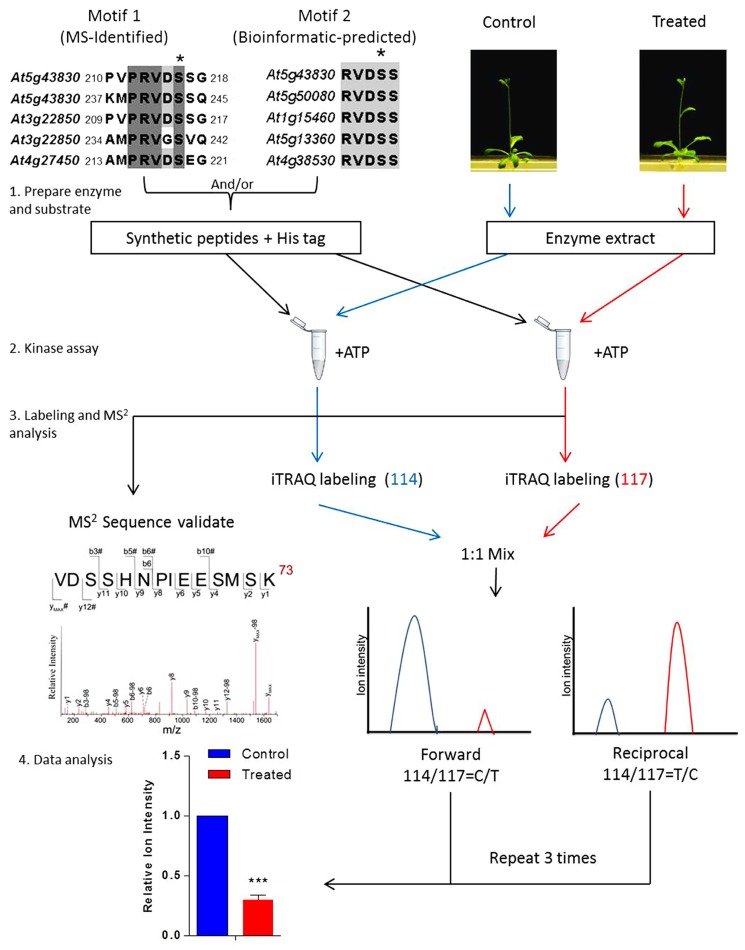
**Workflow of *in vitro* assay for signal-regulated phosphorylation**. Upper left panel shows two alignments of conserved sequences of phosphorylation motif generated with CLUSTAL W, which was reported in a previous study (Li et al., 2009). In this case, 9–21 amino acid-long oligopeptide sequences deduced from the primary sequence of an aluminum-induced protein covering the entire phosphorylation motif was employed to search against the non-redundant protein sequence database (organism: *Arabidopsis thaliana*, taxid: 3702). Candidates with a homology of more than 55.5% are considered to contain the phosphorylation motif. The most conserved candidates are aligned to obtain the motif sequence. The phosphorylation site Ser(S) is marked with a black asterisk and TAIR IDs of the genes are shown on the left. To validate such a prediction, peptides including those amino acid sequences flanking the predicted phosphosite motif are synthesized and used as substrates for *in vitro* kinase assay. The control or treated means the plant without or with particular treatment of interest, respectively. The quantitation is performed at MS^2^ spectra by directly comparing the reporter ion intensity. Plant extracts from the control or the treated plants are used separately. Peptides assayed are reciprocally labeled with iTRAQ reagents. Three sets of reciprocal labelings are required before a statistical analysis is performed.

To place the newly identified phosphosites in the context of a cellular process or a specific signal regulation, a general approach has been developed using quantitative proteomics in combination with kinase extracts of both the treated and mutant plants (**Figure 1**; Li et al., 2009, 2012; Zhu et al., 2012). The *in vitro* kinase assay is then performed using the synthetic peptides mixed with plant kinase extracts in the presence of phosphatase inhibitors. The chemical labeling quantitative proteomic protocol, such as iTRAQ, is then applied to investigate the differentially regulated phosphorylation events. A routine iTRAQ-based *in vitro* phosphosite quantitation requires at least two sets of forward and reciprocal experiments. Results on MS-derived and bioinformatics-derived synthetic peptides can provide information on the differential regulation of phosphosites in response to the internal and external stimuli.

## KINASE/PHOSPHATASE AND PHOSPHOSITE-CONTAINING PEPTIDE INTERACTOMICS

The highly conserved amino acid sequence or phosphosite motif is useful when one wants to isolate the phosphosite-containing peptide-binding proteins. To achieve that, synthetic peptides that have been validated by *in vitro* kinase assay will be used in combination with the ^15^N-stable isotope labeled plants. By this approach, the validated phosphopeptides together with their corresponding non-phosphorylated peptide cognates will be synthesized chemically and conjugated with biotin according to the method described previously (Christofk et al., 2011). Each set of biotinylated peptides will be immobilized on beads and packed into a chromatography column. Light nitrogen-labeled proteins will be passed through a column immobilized with the phosphorylated peptides, whereas the heavy nitrogen-labeled proteins will be passed through a column immobilized with its corresponding non-phosphopeptide. As a result, ^15^N-coded putative kinases and phosphosites-binding proteins are presumably enriched on the column with non-phosphorylated synthetic peptide containing a phosphosite or motif sequence. The two protein passages will be mixed together at 1:1 ratio (w/w) and resolved on SDS-PAGE gel, which will then be tryptic digested and subject to MS analysis as described before (Li et al., 2009; Guo and Li, 2011). Both phosphosite-containing peptides (non-phosphorylated and phosphorylated)-binding proteins will be identified differentially due to a difference in between the heavy and light isotopolog envelopes of their peptide ions.

## *IN VIVO* QUANTITATION OF PTM OCCUPANCY

**FIGURE 2:**
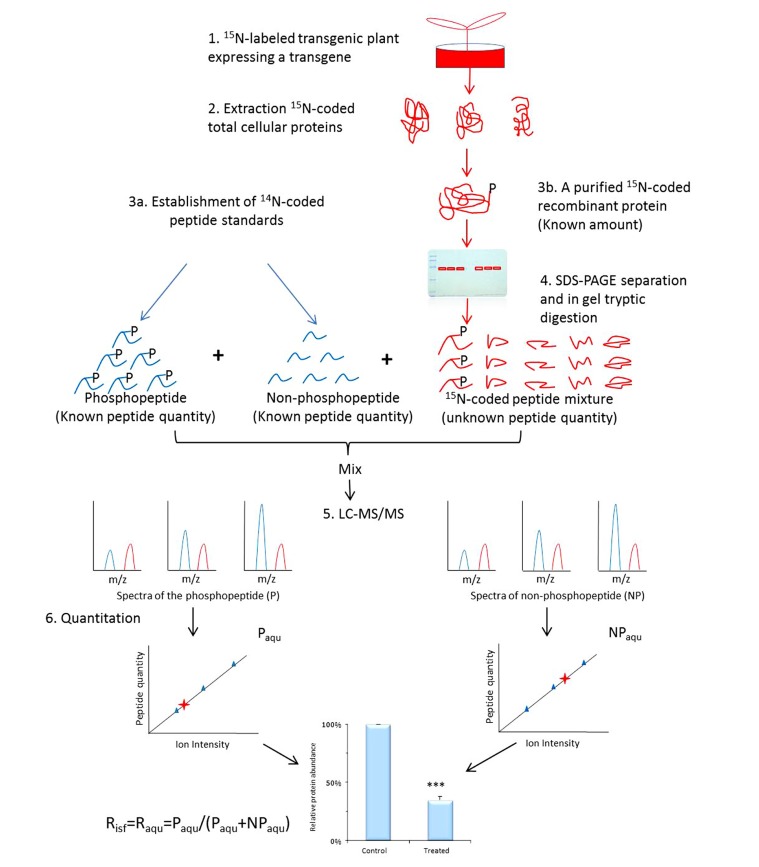
**Workflow of determination of occupancy (*R*_aqu_ or *R*_**isf**_) using AQUIP**. A known quantity of ^15^N-coded recombinant proteins, including both the target phosphorylated isoform and all of its cognates of different types of PTMs, are highly purified from ^1^^5^N-labeled transgenic plant (either the control or the treated plants) and divided into six aliquots and resolved on SDS-PAGE gel, followed by in-gel trypsin digestion. The ^14^N-coded synthetic phosphopeptide and its non-phosphorylated cognate are mixed together with a known concentration and spiked into 6 aliquots of ^15^N-coded peptide samples. After the oxidization of methionine residues on the peptides and LC-MS/MS analysis, two standard curves are built using a method between the ion intensity ratios of peptide *P* and *NP* standards and the molar ratios of peptide standards. The molar amount of phosphorylated peptide (*P*_aqu_) and its non-phosphorylated cognate (*NP*_aqu_), which are derived from the highly purified and ^15^N-coded protein isoforms, is determined according to these two standard curves separately. The percentage of target-phosphorylated isoform among all isoforms in the total cellular proteins is defined as the site-specific phosphorylation occupancy *R*_isf_, which is equivalent to *R*_aqu_ when the ratio of *P* and *NP* peptide amount is in concern (Li et al., 2012). Error values indicate standard deviations. The *R*_isf_ obtained from either the control or the treated plants is used to calculate the relative abundance of the phosphorylation event and subject to statistical assessment, as shown at the bottom of the figure. ***Means significant difference.

Once phosphosites are identified from SILIA-based phosphoproteomics and confirmed with *in vitro* kinase assays, an *in planta* validation and measurement of these phosphosites and signal-specific phosphorylation level will be performed using a transgenic approach. A transgene encoding the target protein will be transferred into plant to measure the alteration of a particular phosphorylation in response to various treatments, cell types, or disease models (Havlis and Shevchenko, 2004; Steen et al., 2008). The term occupancy (*R*_aqu_ or *R*_isf_) defines the ratio of the amount of a protein PTM isomer (or PTM peptide) over the total amount of the corresponding protein (or the corresponding PTM site peptide; Li et al., 2012). A signal-induced alteration in occupancy on a phosphosite usually measures the change in phosphorylation level elicited by both internal and external signals. It is therefore that *R*_aqu_ or *R*_isf_ are used to describe signal-specific phosphorylation change. To determine *R*_aqu_ (or *R*_isf_) of a phosphosite, ^14^N-coded peptide standards are established (**Figure 2**) against the ^15^N-coded recombinant protein expressed in the transgenic plant. One is the synthetic phosphopeptide that contains targeted phosphosite, whereas the other is its unmodified cognate. A series of synthetic peptide samples with a wide range of concentrations are then made, in the meantime, an aliquot of known amount of highly purified recombinant protein expressed in transgenic plant is resolved on SDS-PAGE and undergo in-gel tryptic digestion. Once the peptides are produced from the ^15^N-coded recombinant protein, ^14^N-coded standard phosphopeptides (both phosphorylated and non-phosphorylated) of various amounts are added into aliquots of the recombinant protein-derived peptides and analyzed using MS. The resulting data is used to establish two individual standard curves to calculate the molar amount of phosphopeptide *P*_aqu_ and its non-phosphorylated cognate *NP*_aqu_. The *R*_aqu_ is determined using Eq. 1:

$$\begin{array}{cc} R_{aqu}=P_{aqu}/(P_{aqu}+NP_{aqu}). & \left(1\right) \end{array}$$

Because it is believed that the fold increase from a partial to a full phosphorylation status may lead to different cellular effects (Steen et al., 2008; Wu et al., 2011), a number of MS-based methods have been invented for quantitation of phosphorylated isoform. Using stable isotope-labeled synthetic phosphopeptides and artificial concatemer of standard peptides (Gerber et al., 2003; Mayya et al., 2006; Wu et al., 2011) and QconCAT (Beynon et al., 2005; Anderson and Hunter, 2006; Pratt et al., 2006; Rivers, et al., 2007) approaches are frequently used for quantitative analysis of protein phosphorylation. Since peptide digestion may be incomplete or proteins of interest requires isolation (Brun et al., 2007), protein standard absolute quantification approach, which uses the full-length stable isotope-labeled protein as standard, becomes popular in quantitation of non-PTM proteins (Brun et al., 2007; Lebert et al., 2011). To address the difficulty in measuring abundance of a phosphorylated isoform, AQUIP (**Figure 2**; Li et al., 2012) strategy, which combines these advantageous features with a recombinant HisTag fusion protein (His_8_-BCCP-His_8_), was invented to achieve absolute quantitation for the phosphorylation level of a particular phosphosite *in vivo*. In short, in AQUIP: (1) both ^14^N synthetic peptide standards and purified ^15^N-coded protein of interest are used to determine the total amount of the target protein (*T*_isf_); (2) a pair of synthetic peptides of phosphosites are used to calculate the phosphosite occupancy (*R*_aqu_ or *R*_isf_), and (3) the absolute amount of phosphorylated isoform *P*_isf_ is calculated *via *an equation: *P*_isf_ = *T*_isf_ ∙ *R*_aqu_, where *T*_isf_ stands for the molar amount of the whole recombinant protein isolated from plant cell lysate.

To determine the absolute amount of the recombinant protein in the cell lysate, the plant is first labeled *via* SILIA. The ^15^N-coded total cellular protein is extracted and resolved on SDS-PAGE. The gel slice that contains the targeted protein is excised out to perform the in-gel trypsin digestion and then divided into seven equal fractions. Two gradients of ^14^N-peptide standards of known concentrations are mixed with the target protein sample to establish two standard curves between the peptide concentration and the ion intensity of the whole isotopic envelope. These standard curves established above are used to calculate the molar amount of the ^15^N-coded target peptide from recombinant fusion protein sample. Because the digestion efficiency and recovery rate of each peptide varies during peptide preparation, peptide yield (*km*) of each peptide needs to be determined. To obtain *km*, a highly purified target protein with known amount is obtained *via* tandem affinity purification (Li et al., 2012). The purified targeted recombinant protein is divided into two fractions with high or low molar amount and mixed with equal amount of standard peptides mixtures. The two mixtures of ^15^N-coded target proteins and ^14^N-coded peptide standards are then subjected to protease digestion separately and subjected to MS analysis to build standard curves. These MS results are used to determine the peptide yield *km*. Finally, *T*_isf_ of the targeted protein could be obtained follow Eq. 2:

$$\begin{array}{cc} T_{isf}=\frac{1}{m}\,\Sigma \,(\frac{T_{1}}{K_{1}}+\frac{T_{2}}{K_{2}}\cdot \cdot \cdot +\frac{T_{m}}{K_{m}}\,), & \;\;\;\;\;\;(2) \end{array}$$

where *k* represents peptide yield and *m* represents number of peptides used for quantitation. The absolute molar amount of the PTM isoform is determined as the following Eq. 3:

$$\begin{array}{cc} P_{isf}=T_{isf}\cdot R_{aqu}. & \;\;\;\;\;\;\;(3) \end{array}$$

This method has been applied in Arabidopsis to measure phosphorylation event on transcriptional factor ERF110 in both air-treated and ethylene-treated transgenic plants, and confirmed an ethylene down-regulating phosphorylation event on ERF110 Ser62 in the *ein2-5* mutant background (Li et al., 2012). Its application could be extend to different treatments and plant species, and not limited to quantitation of phosphorylated isoforms. The absolute amount of PTM isoforms could also serve as biomarkers that correlate with phenotypic changes, which could be applied to the clinical aspects (Schrohl et al., 2003; Barnidge et al., 2004).

## *IN VIVO* VALIDATION OF BIOLOGICAL FUNCTION OF A PHOSPHOSITE OR A PHOSPHOPROTEIN

To study the biological function of a phosphoprotein identified from quantitative PTM proteomics, loss of function lines of these phosphoproteins can be obtained by both T-DNA insertion (Krysan et al., 1999) and RNA interference (Klink and Wolniak, 2000) to study the function of these candidate genes *in vivo*. Alternatively, the candidate phosphoprotein gene can be expressed ectopically to monitor the gain-of-function effect of these genes. To further confirm the biological function of phosphosites, phosphorylation- and dephosphorylation-mimetic mutants are introduced to plants. A successful example is that phosphorylation of Ser (S) can be functionally substituted by Asp (D) in a rat liver kinase, resulting in an identical substrate dependency. In contrast, Ala (A) mutant showed exactly the same kinetic property of its de-phosphorylation form (Kurland et al., 1992). These molecular genetic methods have been successfully applied in a wide number of organisms, which include, for example, determination of the phosphorylation-dependent kinase activity of eukaryotic elongation factor 2 kinase (eEF-2K; Tavares et al., 2012), alteration of conformation of human oncogene stathmin by phosphorylation (Curmi et al., 1994) as well as the phosphorylation-dependent accumulation/degradation of both ACC synthase (Liu and Zhang, 2004) and signaling component EIN3 (Yoo et al., 2008) in Arabidopsis. The commonly used amino acid substitution that are mimetic of the dephosphorylation status at phosphosite are amino acid S (or T) mutated to A (or I) and Y to F, respectively. In contrast, the phosphosite (S) mimicking the phosphorylated status is usually mutated to D or E. The mutant genes of site-directed point-mutations are then transformed into a plant to verify its biological function *in vivo* using the wild-type gene as a control. Phenotypes of all three transgenic plants expressing the mutants and wild-type gene reveal the possible role of the phosphosites in plant. By this strategy, the Ser62 phosphosite of ERF110 has been found to play a role in the organ development in Arabidopsis (Li et al., 2012; Zhu et al., 2012).

## CONCLUSION

In conclusion, we have summarized an integrated quantitative and functional phosphoproteomics approach, which starts from a large-scale identification of phosphopeptides, to bioinformatics-prediction, to *in vitro* validation of phosphosites, protein and phosphopeptide interactomics, to *in planta* quantitation of phosphosites and finally to the investigation of biological functions of these phosphosites *in vivo*. Knowledge generated *via* such a strategy might ultimately be integrated to interpret the combinatorial codes of different PTMs (including phosphorylation) events and those PTM changes in response to developmental cues and environmental stimuli. During application of this quantitative PTM proteomics approach to a special biological problem, one may have to pay special attention to the amount of labor involved, the cost for synthesis of peptides and computational skills used in analysis of these large set of MS data.

## Conflict of Interest Statement

The authors declare that the research was conducted in the absence of any commercial or financial relationships that could be construed as a potential conflict of interest.
